# Ubuntu philosophy as a strategy to promote access to under-five child healthcare services

**DOI:** 10.4102/curationis.v48i1.2667

**Published:** 2025-06-11

**Authors:** Livhuwani Tshivhase, Idah Moyo

**Affiliations:** 1 Department of Health Studies, College of Human Sciences, University of South Africa, Pretoria, South Africa; 2 Population Solutions for Health, Harare, Zimbabwe

**Keywords:** access, barriers, children under five, strategy, ubuntu

## Abstract

**Background:**

Inaccessibility of healthcare services for children under five contributes to child morbidity and mortality in sub-Saharan Africa. Children are reportedly dying at home from treatable conditions, hence the need for this study.

**Objectives:**

The study aimed to exploring and synthesising the barriers in accessing under-five child healthcare services through the lens of ubuntu philosophy in sub-Saharan Africa.

**Method:**

An integrative literature review was conducted. Several databases were searched utilising a combination of phrases such as: ‘access’, ‘barriers’, ‘child healthcare services’ and ‘sub-Saharan Africa’. Qualitative and quantitative studies, published between 2014 and 2024 in sub-Saharan Africa, were used.

**Results:**

Study findings revealed that there were parental- or guardian-related factors, healthcare provider factors and healthcare environment factors that can hinder access to healthcare services for children under the age of five.

**Conclusion:**

Intervention measures to address the barriers to access of healthcare services by children under five should embed ubuntu values by all healthcare providers and policymakers. Continuous health education to empower parents and guardians on childcare practices is recommended.

**Contribution:**

The study provides insights into challenges of accessing child healthcare services. These findings are key for nurse managers, nurse educators and policymakers to better plan for comprehensive quality service provision. Leveraging on Ubuntu philosophy would be pivotal to making a critical analysis of these challenges and how to address them.

## Introduction

Ubuntu is an African philosophy that denotes the idea that a person’s existence is intrinsically linked to the existence of others. According to Mayaka and Truell (2021), ubuntu entails universal life values inclusive of love, respect and reciprocity, equality and justice, integrity and accountability, caring and trust, unselfishness and social change. Ubuntu philosophy is central to the protection of children through families and community networks. The philosophy has the potential to provide children with protection, counselling, support and mentorship in an African context as well as informing ideal child-rearing practices (Mugumbate & Chereni 2019). Tutu (1999) stated that ‘ubuntu means to be generous, hospitable, friendly, caring and compassionate’ (Shambare 2021).

Ubuntu philosophy provides the basis for childcare and child protection through immediate family, extended family, faith-based organisations and the community in general. The implications, therefore, support the saying that ‘it takes a community to raise a child’. Ubuntu philosophy aligns well with the indigenous African beliefs that a child belongs to the entire community, not only to the immediate family (Akintayo 2021; Isidienu 2015; Olaore & Drolet 2016). Integrating ubuntu philosophical values into the integrated management of childhood illnesses (IMCIs) could help prevent most of the barriers to child healthcare services access.

Integrated Management of Childhood Illness strategy is a World Health Organization (WHO) and United Nations Children’s Fund (UNICEF) initiative aimed at reducing under-five children deaths (WHO 2023). Among the benefits of the strategy is the improvement of family and community practices to reduce child morbidity and mortality (Boschi-Pinto et al. 2018; Kilov et al. 2021; Jensen, McKerrow & Wills 2019; Mulaudzi 2015). Sub-Saharan Africa has the highest under-five mortality in the world, as 13 children die before their fifth birthday (WHO 2023; Tefsa et al. 2021). The healthcare providers are trained to enhance the implementation of the strategy with the aim of improving child healthcare services. Although the IMCI strategy is regarded as the best in reducing under-five child mortality and morbidity, there are barriers for parents and caregivers in accessing such care. Caregivers and parents with ill children are to access healthcare from healthcare facilities timeously before children’s health deteriorates.

The improvement of access to maternal and child healthcare services is the first step in bringing down the rates of newborn and child mortality (Handebo et al. 2023). This could lead to quality child healthcare services and could reduce child mortality as well as assist countries in the attainment of the Sustainable Development Goal 3 on health and well-being. Most caregivers were reported to be failing to get access to child health services due to long queues and poor staff attitudes by healthcare providers (Madhi et al. 2020; Profitt et al. 2023). Such poor staff attitudes are an indication that ubuntu philosophy is not applied by healthcare providers. Some caregivers, including parents, were choosing to travel long distances to seek child healthcare where they thought it is available and affordable (Izugbara & Wekesah 2017; Meyer, Oppong Asante & Lukobeka 2018; Tshivhase et al. 2024). Getting treatment from faraway facilities could have been taxing to some parents who could have been poor. These barriers to accessing childcare services could easily be overcome if all the healthcare providers as well as those in leadership or management positions had ubuntu values driving them to care. Health seeking is preceded by a decision-making process, which is influenced by individual behaviours, community norms, expectations and provider characteristics and behaviours (Olenja 2003 cited in Handebo et al. 2023). The behaviours of the providers as well as community norms can act as barriers to accessing the care and could be interpreted as against the ubuntu philosophical values. Ubuntu values encompass love, acceptance, respect and sharing (Netshisaulu & Makhema 2021). The ubuntu values among healthcare providers could thus enhance humanness and improve their poor attitudes towards parents and guardians of children under-five accessing healthcare services.

Health seeking in children under five is unique because it is the parent or the caregiver who decides upon the type and frequency of healthcare services utilisation. Many mothers and caregivers do not seek medical care for their children (Abegaz, Berhe & Gebretekle 2019). For children, whether to access healthcare and from where to access, it is dependent upon a parent or a caregiver. That decision is determined by the availability of healthcare services as well as social and economic factors (Handebo et al. 2023).

Some studies conducted in India and elsewhere (Bennet et al. 2015; Gupta et al. 2020; Ismail, Iqbal & Nasr 2019) indicated that cultural, social and economic factors were influencing caregivers in accessing childcare services. The same studies reported that caregivers were seeking child healthcare services with a priority for male children. Such discriminatory acts are an indication that ubuntu philosophy is not valued as a human right for equal treatment for all (Goldstone 1997). In Tanzania, such discrimination does not exist, as they treat both genders equally (Kanté et al. 2015). The nondiscriminatory practice aligns well with the ubuntu values, as all human beings are equal before the law. The practices of gender discrimination are an indication that the ubuntu values that regard humans as equals are disregarded and need to be challenged through policies and strategies that incorporate ubuntu values. Some barriers to accessing child healthcare are related to the educational status of caregivers or mothers. Educated mothers and caregivers are reported to seek healthcare for common childhood illnesses than their uneducated counterparts, who might not even recognise the symptoms of illness (Ahinkonah et al. 2021; Amofa 2020; Nu et al. 2020). This implies that the educational level of the parents is also necessary for them to comprehend the importance of accessing under-five child healthcare services.

Some caregivers and parents fail to seek healthcare due to community barriers such as tension around communication and trust between caregivers and providers, high patients load, medicines stock outs and not having 24-h staffing (Kilov et al. 2021). Such barriers could be reduced through the implementation of ubuntu philosophy, as Chigangaidze, Matanga and Katsuro (2021) indicated that ubuntu empowers children, families, communities and workers. Furthermore, healthcare providers with ubuntu could be generous, hospitable, friendly, caring and compassionate, as ubuntu values shared by Tutu (1999). Such providers with the ubuntu values could thus be compassionate and generous to provide accessible child healthcare services. It is against this background that the researchers sought to explore and synthesise barriers to accessing under-five child healthcare services through the lens of ubuntu philosophy in sub-Saharan Africa.

### Aims of the review

The study aimed at exploring and synthesising the barriers to accessing under-five child healthcare services through the lens of ubuntu philosophy in sub-Saharan Africa.

## Research methods and design

Researchers conducted an integrative review. Depending on the empirical or theoretical literature, integrative reviews are considered as an appropriate approach for the review purpose (Whittemore & Knafi 2005). The review effectively helps researchers to fully understand the barriers in accessing under-five child healthcare services. Integrative reviews are good in reviewing, critique to resolve inconsistencies in the literature and provide fresh, new perspective in a topic of the study (Toracco 2016). The researchers followed the methodology suggested by Whittemore and Knafl (2005) as cited in Vagharseyyedin (2016) Jun, Kovner and Stimpfel (2016). The steps followed in this review were problem identification, literature search, data evaluation, data analysis and presentation of the findings. The researcher’s choice of integrative review was for its ability to explore published evidence regarding certain phenomenon, thereby identifying potential research gaps that need to be addressed during subsequent research.

### Literature search strategy

A literature search on barriers to accessing healthcare for children under the age of 5 was done by using the Boolean ‘and/or with keywords such as ‘access and mother’, ‘barriers and mothers’, ‘access and barriers’, ‘childhood illnesses and challenges’, ‘access and child health’, ‘positive experiences and access’. and so on. Eight databases were searched (EBSCOhost; Science Direct; PubMed; Medline; Clinical key; Cochrane; Wiley and Ovid/ LWW health Library) with the assistance of a skilled librarian. Some literature was searched for from the reference lists of the other relevant articles used. Peer reviewed articles were used in the integrative review, but grey literature was excluded. A total number of 466 articles were imported into Endnote, including articles that were identified through manual searches after excluding articles by examining the title.

#### Inclusion criteria

**FIGURE 1:**
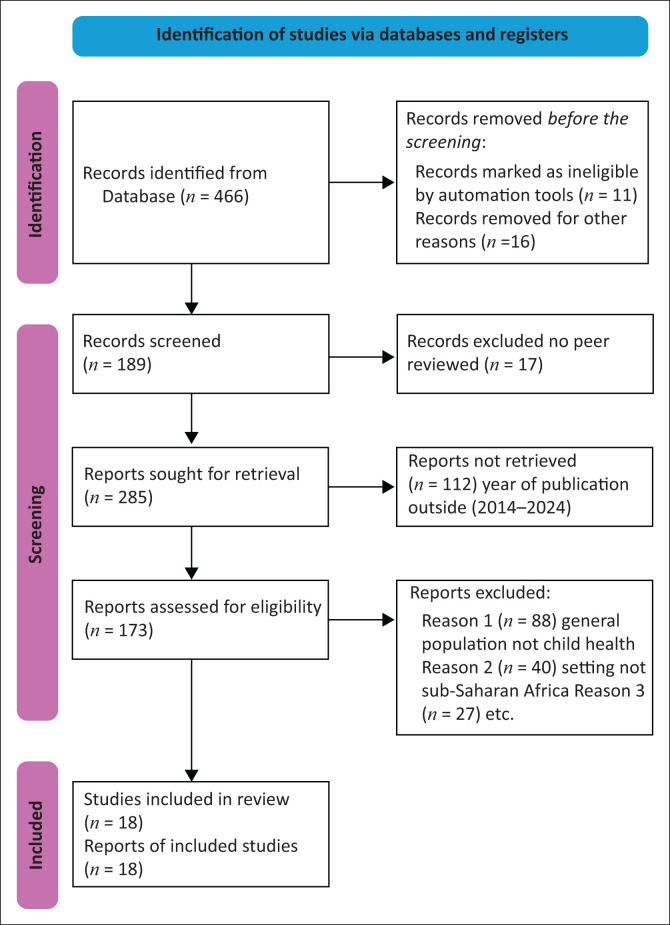
Flow chart indicating the selection criteria of articles reviewed.

The inclusion criteria were as follows: published studies that were primary research articles, focusing on barriers and facilitators in accessing child health and written in English. In addition, the original research studies must have been carried out in the last 10 years from 2014 to 2024. Only studies conducted in sub-Saharan Africa were included. Systematic reviews and scoping review articles covering the same title were also included. Figure 1 presents the flow chart of the reviewed articles for the study.

#### Exclusion criteria

Grey literature such as thesis and dissertations, conference reports, poster, abstracts and blogs were excluded on the basis that they were not peer reviewed.

### Data evaluation

Data evaluation was done by the reviewers who critically judged whether the data elements or results were worth remaining in the study data set. Evaluation was done to increase the quality of sources in the review methods and address the primary sources meaningfully (Whittemore & Knafl 2005). L.T. and I.M. read and evaluated the 466 articles for possible duplication, relevance, methodology, quality of samples and sampling methods. An independent reviewer assessed the quality of all articles included in the review. Both authors collectively read and discussed the reviewed articles until reaching consensus on the eligible articles. Eighteen articles met the inclusion criteria. Critical appraisal skill programme (CASP) studies were followed in the evaluation of articles selected in the reviews (CASP 2013a). A checklist was used for data evaluation and is presented in Table 2 which presents the CASP for qualitative studies and Table 3 which presents the CASP for quantitative studies (Bowling 2009; Pearson 2004).

#### Data analysis

The authors read the interpretative descriptions and summaries of results from the 18 selected studies independently. The summaries of the data were sorted thematically to synthesise and identify the key emerging issues as guided by Whittemore et al. (2014). Similar findings were grouped together as themes and then into categories as articulated by Whittemore et al. (2014) and Whittemore & Knaff (2005). The authors met to discuss their individual coding processes as knowledgeable researchers. The process yielded three themes agreed upon which reflect major issues relating to access to childcare services in sub-Saharan Africa. This is according to postulations by some authors (Allen et al. 2017; Lungu et al. 2016; Madhi et al. 2020; Nyande et al. 2024).

### Ethical considerations

This article followed all ethical standards for research without direct contact with human or animal subjects.

## Results

### Study demographics

A total of 18 articles were included in the review. The review was limited to sub-Saharan Africa. Most studies were qualitative (*n* = 14), followed by quantitative (*n* = 2) and systemic review (*n* = 2). The distribution of the study by countries was as follows: systematic review (*n* = 1) and scoping review (*n* = 1) were conducted in sub-Saharan Africa; Ghana (*n* = 1); Kenya (*n* = 1); Ethiopia (*n* = 2); South Africa (*n* = 6); Malawi (*n* = 3); Somali (*n* = 1); Uganda (*n* = 1) and Nigeria (*n* = 1). Table 1 presents a summary of the articles reviewed in this review. Table 2 presents the qualitative studies appraisal checklist, while Table 3 presents the quantitative studies appraisal checklist as outlined by Pearson (2004) and Bowling (2009), respectively.

**TABLE 1:** Summary of articles included for the review.

No.	Author	Setting	Sample	Methods or research design	Purpose of the study	Findings
01	Profitt et al. 2023	South Africa (Cape Town)	62 community members	Exploratory mixed method	To uncover the access barriers and facilitators underlying the high burden of out-of-hospital deaths.	Barriers to access child healthcare services were cited as: long waiting times, staff attitude, transport challenges, language barriers, cost of healthcare and fears of accessing care.
02	Madhi et al. 2020	South Africa	503 randomly selected caregivers for children 0–59 months	Cross-sectional survey	To examine patterns of child healthcare uptake and barriers that affect access to healthcare in two South African (SA) low-income urban settings.	Barriers to accessing child healthcare services were long queues at health facilities. poor attitudes of healthcare personnel; lack or shortages of medicines and long distance to healthcare facilities.
03	Adugna et al. 2020	Sub-Saharan Africa	15 articles scoping review	Scoping review	To discover the barriers and facilitators to healthcare access for Children with Disability (CwD) in selected low to middle income sub-Saharan African countries	Major barriers included stigma and negative attitudes, poverty and insufficient resources, inadequate policy implementations, physical inaccessibility, lack of transportation, lack of privacy and inadequately trained healthcare professionals to deal with disability.
04	Lungu et al. 2016	Malawi	100 participants with 89 in focus group discussion and 11 on in-depth interviews with caregivers of children under 5 years and health providers.	Qualitative design	To explore healthcare-seeking practices for common childhood illnesses focusing on use of biomedical health services and perceived barriers to accessing under-five child health services in urban slums of Lilongwe, Malawi’s capital city.	Barriers reported were: staff rudeness and uncaring behaviours, lack of medicines and supplies, superficial assessment of child, inappropriate examination of the child, negative attitudes of some health workers, lack of interpersonal skills among health workers.
05	Haskins et al. 2017	South Africa (KZN)	Focus group discussions with community members	Qualitative explorative descriptive design	To generate knowledge about the role of and influences on, caregivers with regard to decision-making about when and where to seek care for sick children	Seeking childcare from traditional healers. Lack of persons to assist children, grandmother as final decision maker.
06	Tshivhase et al. 2024	South Africa (Limpopo)	16 participants who were guardians to children under five	Interpretative phenomenology (Qualitative)	To explore the barriers to accessing and utilising under-five PHC (Primary Health care) services in the Vhembe District.	Common barriers experienced by the participants were long distance to healthcare facilities, lack of resources, long waiting times, poor time management and healthcare provider’s lack of commitment and work devotion.
07	Fekadu et al. 2024	Ethiopia	Six Focus Group Discussion (FGD) and 20 interviews with mothers and caregivers of children under five	Qualitative designs	To identify barriers to equity in the use of child healthcare services in Ethiopia.	Child healthcare barriers were reported as: low-socioeconomic status, geographical inaccessibility of healthcare facilities, community perception and cultural restrictions, political instability and conflicts in the country as well as closure of healthcare facilities during working hours
08	Ustrup et al. 2014	Malawi	1669 caretakers of children under 5 years	Quantitative design	To explore demographic and socioeconomic barriers to healthcare for childhood illnesses and assessing the direct and indirect costs of seeking care.	Respondents cited long distances to healthcare facilities, financial barriers and poor road conditions, high travel cost, long distances, cultural attitudes and beliefs of the population as barriers to accessing children healthcare services.
09	Nyande 2024	Ghana	10 nurses and nine caregivers of children under 5 years	Qualitative study	To explore the experiences of nurses and caregivers about the health system bottlenecks to the delivery of child healthcare services in a rural district.	Hinderances to access child healthcare services were shortage of nurses, inexperienced nurses, undesirable care services and attitude displayed by nurses.
10	Allen et al. 2017	Uganda	36 focus groups with caretakers of children under 5 years	Qualitative design	To explore challenges to seeking healthcare for children under five in Uganda.	Caretakers raised geographical access barrier, facility management barrier, facility commodities, staffing, financial circumstances and domestic conflicts as contributory barriers to accessing under-five childcare services.
11	Adedini et al. 2014	Nigeria	18 028 women who gave birth to children under 5 years	Quantitative design	To determine substantial barriers to accessing child healthcare in Nigeria.	Raised challenges in accessing childcare services were: difficulty in getting money, poor interaction with healthcare workers, inadequate supply of drugs and shortage of healthcare workers.
12	Mohamed et al. 2021	Somali	17 in-depth interviews and seven focus group discussions and field observers with lactating/pregnant mothers and healthcare providers and traditional birth attendants and camp leaders	Qualitative design	To understand barriers to the use of maternal and child healthcare services among internaly displaced people (IDP) in Mogadishu.	Several barriers were cited and are: low-socioeconomic status barriers; lack of decision-making power of women; fear of going to unfamiliar areas; traditional beliefs; male dominance in decision making; lack of family support; lack of privacy in the facility; transportation challenges, poor functional services, closure of some facilities in some hours, lack of referral pathways; negative experiences from facilities.
13	Kaunda et al. 2021	Malawi	19 participants (clinical, nurses, drivers, mothers and a father)	A qualitative descriptive	To assess facilitators and barriers to referral of children under the age of 5 years at Ndirande Health Centre in Blantyre, Malawi.	Participants reported the following barriers: Lack of transport, lack of basic requirements on the availability of ambulance; facilities far from reach, parental beliefs and the cost of referrals to the healthcare facilities which were expensive.
14	Okondo et al. 2022	Kenya	94 women and 48 men	Qualitative design	To understand the experiences of care for parents or caregivers (caregivers) as they navigate the hospital system with the aim of identifying opportunities to improve service delivery and child health outcomes.	Parents indicated that they did not like to take children to health facilities due to long waiting times, overcrowded unhygienic conditions of the facilities and strict visitation policies as well as confusing payments for child healthcare services.
15	Acheampong, Lowane & Fernandes 2023	South Africa	18 migrant mothers	Qualitative design	To explore the experiences of migrant mothers in utilising child immunisation services in primary healthcare facilities.	Participants who happened to be migrant mothers indicated the following as hinderances to accessing child healthcare: difficulty in communicating with the healthcare workers because of language barriers, access challenges, interpersonal barriers and interpersonal relationships which were poor.
16	Sidze et al. 2022	Sub-Saharan Africa	53 research articles	Systematic review (Qualitative and Quantitative studies)	To share the results of a systematic review on the state of inequalities in access to and utilisation of maternal, newborn and child health (MNCH) services in the sub-Saharan African region.	Common barriers raised were distance to health facility, availability of quality service, discriminatory attitudes of healthcare personnel, low level of education and unemployment.
17	Mkabile & Swartz 2022	South Africa	20 parents and primary caregivers of children	Qualitative design	To explore the caregivers’ experiences regarding the difficulties in accessing childcare services.	Parents reported about the struggles with transport, crowded, dangerous and hostile environments that made accessing child healthcare difficult.
18	Mengistu et al. 2021	Ethiopia	664 participants, 60 focus group discussions and 60 in-depth interviews	Qualitative design	To assess mothers or caregivers’ healthcare seeking behaviour for their children in Northwest Ethiopia.	Participants reported failing to access child healthcare services for the following reasons: Misconceptions about illness causation, compounded by preference for traditional healers has affected service uptake, health post closure and drug stock-out led to inconsistent availability of services; limited confidence and skill among health extension workers and under-resourced physical facilities.

Note: Please see the full reference list of this article, https://doi.org/10.4102/curationis.v48i1.2667, for more information.

FGD, Focus Group Discussion; CwD, Children with Disability; PHC, Primary Health Care; IDP, Internally Displaced People.

**TABLE 2:** Critical appraisal checklist of the qualitative studies.

Criteria	Yes	No
1. Congruity between stated philosophical perspective and research methodology	14	2
2. Congruity between methodology and research question or objective	14	2
3. Congruity between methodology and methods used to collect data	16	0
4. Congruity between methodology and representation and analysis of data	14	2
5. Congruity between methodology and interpretation of results	16	0
6. There is a statement locating the researcher culturally or theoretically	5	11
7. The influence of the researcher on the research and vice versa is addressed	14	2
8. Was the data analysis sufficiently rigorous?	12	4
9. Ethical issues taken according to current criteria, and evidence of ethical approval.	16	0
10. Conclusions drawn from analysis or interpretation of data	12	4

*Source*: Adapted from, Pearson, A., 2004, ‘Balancing the evidence: Incorporating the synthesis of qualitative data into systematic reviews’, *JBI Evidence Implementation* 2(2), 45–64

**TABLE 3:** Critical appraisal checklist of the quantitative studies.

Criteria	Yes	No
1. Aims and objectives clearly stated	2	0
2. Hypothesis or research question clearly specified	2	0
3. Dependent and independent variables clearly stated	1	1
4. Variables adequately operationalised	0	2
5. Design adequately described	2	0
6. Method appropriate	2	0
7. Instrument used tested for reliability and validity	2	0
8. Sample, inclusion or exclusion and response rate described	1	1
9. Ethical consideration	2	0
10. Was the study piloted?	2	0
11. Statistical analysis appropriate	2	0
12. Results reported and clear	2	0
13. Conclusions do not go beyond limit of data and results	2	0
14. Results reported related to hypothesis and literature	1	1
15. Limitations reported	2	0
16. Conclusions do not go beyond limit of data and results	0	2
17. Findings able to be generalised	2	0
18. Implications discussed	1	1
19. Conflict of interest with sponsor	0	2
20. Data available for scrutiny and re-analysis	2	0

*Source*: Adapted from Bowling, A., 2009, *Research methods in health: Investigating health and health services*, Open University Press, Maidenhead

### Study findings

The analysis of all the 18 reviewed studies yielded three general themes on barriers in accessing under-five child healthcare services in sub-Saharan Africa. A narrative empirical synthesis was done by the researcher as follows: the researchers investigated the similarities and differences between the studies and explored the relationships within the data. The assessment of the strength of the evidence and results from both the studies were summarised as themes based on the question of the study to inform the practice (Lisy & Porritt 2016). Themes are parental or guardian-related factors, healthcare provider factors and healthcare environment factors. The subthemes were sociocultural beliefs; misconceptions about causes of childhood illnesses; lack of family support; low level education, healthcare workers attitudes and behaviours; difficult communication and language barriers; poor interpersonal relationships; stigma, early health post closure; distance to healthcare facility; overcrowded facilities; long waiting times; shortage of resources. Themes and subthemes are outlined in Table 4.

**TABLE 4:** Themes and subthemes for the review.

Themes	Subthemes
1. Parental or guardian-related factors	1.1 Sociocultural beliefs 1.2 Misconceptions about causes of childhood illnesses 1.3 Limited awareness of free curative child services 1.4 Lack of family support 1.5 Low level education 1.6 Low-socioeconomic status
2. Healthcare provider factors	2.1 Healthcare workers’ attitudes and behaviours 2.2 Difficult communication and language barriers 2.3 Poor interpersonal relationship 2.4 Stigma
3. Healthcare environment factors	3.1 Early health post closure 3.2 Distance to the healthcare facility 3.3 Overcrowded facilities 3.4 Long waiting times 3.5 Shortage of resources (stock out, staff)

#### Theme 1: Parental or guardian-related factors

Several reviewed articles highlighted the fact that parental or guardian-related factors affect their ability to access child healthcare services. Some of the parents and guardian’s access to child healthcare services were determined by sociocultural beliefs, misconceptions about causes of childhood illnesses, limited awareness of free curative child services, lack of family support, low level education and low-socioeconomic status (Khan 2019; Zakayo et al. 2020).

Several studies revealed that some of the parents had sociocultural beliefs that restricted their ability to access child healthcare services as some believed in and preferred traditional healers over western medicine (Haskins et al. 2017; Mohamed et al. 2021; Nyande et al. 2024; Ustrup et al. 2014). Some of the parents had misconceptions about the causes of childhood illnesses which made them not access healthcare facilities for child healthcare services (Mengistu et al. 2021; Mkabile & Swartz 2022). Awareness of free curative child services was limited to some parents which made them not access childcare from healthcare facilities though the services were free (Adugna et al. 2020; Profit et al. 2023). Some of the parents lacked family support and as such could not access child healthcare services (Allen et al. 2017; Mohamed et al. 2021). Low level of education was associated with poor access to child healthcare services (Madhi et al. 2020; Mohamed et al. 2021; Sidze et al. 2022). Parents of low-socioeconomic status were found to rely on others to have access to child healthcare services (Adugna et al. 2020; Fekadu et al. 2024; Haskins et al. 2017; Mohamed et al. 2021).

#### Theme 2: Healthcare provider factors

Parents and guardians of children under five cited healthcare provider factors as having the influence in accessing child healthcare services. Healthcare provider factors reflected on literature ranged from their attitudes and behaviours; difficult communication and language barriers; poor interpersonal relationship and stigma as hampering their access to healthcare services.

Attitudes and behaviours of healthcare providers were found to be influencing parental access to child healthcare services. Staff attitudes towards parents of children under the age of 5 years were seen as the reason for parental failure to access child healthcare services (Lungu et al. 2016; Madhi et al. 2020; Nyande et al. 2024; Profitt et al. 2023; Sidze et al. 2022).

Migrant parents who find themselves with sick children around the sub-Saharan African region had difficult communication due to language barriers. These were stumbling blocks for them in trying to access childcare services (Acheampon et al. 2023; Profitt et al. 2023). Some parents found the healthcare facilities inaccessible because of poor interpersonal relationships of clients and healthcare providers (Acheampon et al. 2023; Kaunda et al. 2021; Lungu et al. 2016). The stigma that was attached to parents and guardians by healthcare providers had an influence on the parents and guardians in accessing childcare services (Adugna et al. 2020; Ustrup et al. 2014).

#### Theme 3: Healthcare environment factors

Healthcare environment factors play a vital role in parents and guardians accessing under-five child healthcare services. Several authors cited the following factors as determinants of access among parents and guardians: early health post closure; distance from the healthcare facility; overcrowded facilities; long waiting times and shortage of resources such as stock outs and staff shortages.

Parents could not access child healthcare services where facilities closed earlier before everyone knocked off from work (Mengistu et al. 2021; Mkabile & Swartz 2022; Mohamed et al. 2021). Some facilities were out of reach of parents and not accessible because of the distance, geographical layout of the facilities as well as the lack of transport fees to reach the child healthcare services (Fekadu et al. 2024; Mkabile & Swartz 2022; Profit et al. 2023; Sidze et al. 2022; Tshivhase et al. 2024; Ustrup et al. 2014). Some of the healthcare facilities were inaccessible to parents because of overcrowded facilities in which the waiting time was above three hours accounting for long waiting times (Madhi et al. 2020; Okondo et al. 2022; Profit et al. 2023; Tshivhase et al. 2024).

Parents were discouraged from visiting facilities without enough healthcare providers to give comprehensive childcare services and where there was no treatment and were made to buy from the dispensaries (Adedini et al. 2014; Madhi et al. 2020; Mkabile & Swartz 2022; Kaunda et al. 2021; Nyande et al. 2024).

## Discussion

The integrative review’s purpose was to examine literature and determine the barriers of accessing child healthcare services from the perspective of ubuntu philosophy in sub-Saharan Africa. The region is still battling with mortality rates of children under five and would benefit from the interventions that could be developed from this study. Ubuntu philosophy was applied in all themes as an instrument to curb access challenges for parents and guardians accessing child healthcare in the healthcare facilities. Parents are influenced in accessing healthcare services by their belief systems. Those who believed in traditional medicine delayed seeking healthcare by first starting to explore all that they believed in (Mohamed et al. 2021; Mengistu et al. 2021; Ustrup et al. 2014). Some of the parents or guardians had misconceptions about the causes of childhood illness which resulted in them not seeking healthcare services for childhood illnesses (Mengistu et al. 2021; Ustrup et al. 2014). Chibwana et al. (2009) reported how parents delayed seeking treatment for fever because of trust in traditional medicines and their traditional belief systems. Other parents were unaware that curative child healthcare services were free and failed to access care thinking they needed to pay (Okondo et al. 2022). Capacitation of parents and guardians through health education could thus enhance knowledge of some common childhood illnesses which could then promote nondelay in accessing child healthcare. Individuals who apply ubuntu philosophy in daily life can tackle any conflicts, shift the moral reasoning and ethical decision making in public health and medicine (Sambala, Cooper & Manderson 2020). The statement implies that the ubuntu values among parents and guardians could enable them to seek timeous child healthcare services.

Because accessing child healthcare might mean walking a distance from homes to healthcare facilities, family support was seen as vital in improving access to under-five child healthcare (Allen et al. 2017; Mohamed et al. 2021). Family support was found necessary for accessing healthcare facilities, as parents or guardians may need money for transport, medication buying in case of drug shortages and consultation fees wherein they are expected to pay (Adedini et al. 2014; Allen et al. 2017). Mugumbate and Chereni (2019) note that there are central commonalities with reference to the concept of ubuntu across sub-Saharan African countries despite the different values and practices. In the same vein, utilisation of the ubuntu philosophy could promote support systems in families and communities as the concept emphasise collective responsibility, mutual support as well as caring for each other a point further articulated by Mayaka and Truell (2021).

Studies found that parents of low educational level delayed accessing healthcare because of the lack of the ability to assess the severity of the illness (Adugna et al. 2020; Sidze et al. 2022). In contrast, those of high education level accessed child healthcare services earlier than their counterparts (Tshivhase et al. 2024). Similarly, parents with lower socioeconomic status delayed seeking healthcare services for their children, unlike their higher-status counterparts (Adugna et al. 2020; Fekadu et al. 2024; Mohamed et al. 2021). Ubuntu values such as compassion and love could curb factors such as lack of family support and thereby reduce the barriers to accessing child healthcare. These parental factors of low-socioeconomic status and lack of family support as barriers to access child healthcare services are an indication that the ubuntu values of love and sharing are not applied in our communities. Ubuntu philosophy is rooted on sayings such as ‘it takes a village to raise a child’ and that a person is existent to help the other as an interpretation of an African philosophy that states that ‘muthu ndi muthu nga munwe’ (Mulaudzi & Gundo 2024). Humanness as emphasised in the ubuntu philosophy could encourage communities to work and support each other in accessing child healthcare services.

Healthcare providers with uncaring behaviours and negative attitudes were seen as a threat to the access of child healthcare services by parents (Lungu et al. 2016; Madhi et al. 2020; Nyande et al. 2024; Profitt et al. 2023; Sidze et al. 2022). Some healthcare providers were poorly communicating with parents and guardians of children under five (Acheampon et al. 2023; Profitt et al. 2023). Some of the parents or guardians were migrants who could not speak the local languages, and language became a barrier for them in accessing healthcare (Acheampon et al. 2023; Masciale et al. 2023). Mukuni and Tlou (eds. 2021) concluded that through the application of the ubuntu values, one could enhance the intercultural communication. This implies that communication cannot be a barrier even to migrants if ubuntu is applied when care is provided. Some of the healthcare providers lacked good interpersonal relationship with parents resulting in poor access for child healthcare services (Acheampon et al. 2023; Lungu et al. 2016). The negative attitudes of healthcare providers are an indication that ubuntu values are not considered. Similar findings were reported in a South African study (Nyandeni et al. 2024). The study concluded that incorporating the ubuntu philosophy in the maternal and child health services environment improves the attitudes and respect for patients.

Some parents and guardians who felt stigmatised by healthcare providers saw no need to bring their children for healthcare services (Adugna et al. 2020; Buthelezi, Modeste & Phetlhu 2021). The behaviours and attitudes of healthcare providers are not justifiable as they need to practise ubuntu as they are practising a caring profession. If ubuntu philosophy was part of the values embedded in each of the health professions, healthcare providers were not going to vent their frustrations on parents. Ubuntu and nursing care are interrelated, and they both centralise humanness (Rasweswe et al. 2024). All the healthcare provider barriers such as staff attitudes, and stigma could be addressed by applying the ubuntu values, and the barriers of accessing child healthcare services might be reduced.

The review revealed that parents and guardians of children under five were discouraged from accessing child health services from healthcare facilities for several reasons. Some healthcare facilities closed early before parents could seek healthcare services (Mengistu et al. 2021; Mohamed et al. 2021). Because sub-Saharan regions are mostly rural, most parents were unable to reach the healthcare facilities because of the distance to the facility, poor roads and unavailability of transport to access facilities of child healthcare services (Fekadu et al. 2024; Mkabile &Swartz 2022). This is a clear indication that the status of the region needs the intervention of the governmental stakeholders and policy developers to prioritise healthcare so that it is accessible, available and affordable. Ubuntu application in leadership is necessary as is regarded as a means of demonstrating humanity for others (Mupedziswa, Rankopo & Mwansa 2019). Mutwarasibo and Iken (2019), indicated that ubuntu in leadership is a leading way that prioritise all stakeholders and creates a sense of communication and belonging. Additionally, Jian and Men (2017) as well as Tauetsile (2021), reported that ubuntu leadership created a work environment that fostered employee engagement, job satisfaction and encourage open communication between leaders and their employees. This implies that if ubuntu leadership is in practice, managers responsible for healthcare planning could prioritise child healthcare services.

Parents and guardians revealed that the healthcare facilities were always crowded with patients which discouraged them from seeking childcare from such facilities. Most of the studies highlighted long waiting times which discouraged parents from visiting healthcare facilities (Madhi et al. 2020; Okondo et al. 2022). Those who visited private healthcare facilities commended quick services and timeous appointments that were honoured by healthcare providers than in the public sector healthcare facilities (Ward et al. 2017). Most parents reported shortages of medication for children and shortage of staff to tender care in the healthcare facilities (Madhi et al. 2020; Nyande et al. 2024). This could be the reason behind the unwanted behaviours of the healthcare providers if they were short staffed and lacked equipment to use during their execution of duties. A study by Cataldo et al. (2017) noted that some patients were sent back to access care the following day in circumstances where facilities were short staffed and overcrowded. Such experience of dual visits could thus be a double burden to parents who might be from low-socioeconomic status. Dual visits for parents are an indication that there is still a lack of the ubuntu principles such as love, compassion, sharing as well as respect for another human being. Mangena-Netshikweta et al. (2019), in a study conducted in the South African healthcare, recommended adequate number of well-trained health professionals to every healthcare facility. The recommendation came up as staff members complained of the workload and long waiting times for patients. The ubuntu values, if honoured by all, including by the governmental officers responsible for managing health, was were going to be a catalyst in the acquisition of enough material and human resources to facilitate the smooth running of the healthcare services. Ubuntu means doing to others what you wish to be done to you. Ubuntu is missing and its infusion into our healthcare system could revitalise the system and improve access to our healthcare system. Therefore, incorporating ubuntu could be a vehicle to improve child healthcare services access and aid in the attainment of the sustainable developmental goal for health for all.

### Limitations

The findings of this review provided more insights into understanding access to under-five child healthcare services. However, there are limitations to be considered. Studies that may have been relevant, could have been missed as the review only included those published in English and only those studies that were peer reviewed. The study period was from 2014 and those conducted earlier might have valuable data. Only sub-Saharan Africa setting was included, and other regions might have other valuable data that could have been missed in the reviewed studies. The studies were mostly qualitative in nature and therefore, there may be no generalisability of the study findings. The use of different designs made it impossible to compare the findings.

### Recommendations

Ubuntu philosophy should be part of the healthcare provider training curriculum so that each nursing personnel who practise must have it embedded in each of their clinical skills. Ubuntu concept to be incorporated into IMCIs in-service training for communities and healthcare providers. It is recommended that health education as well as human rights and children rights be strengthened in communities for the communities to revive the ubuntu values. Communities should be empowered with knowledge about causes and management of childhood illnesses to correct the misconceptions that could prevent them from accessing child healthcare services. Government should implement policies of making healthcare accessible and affordable for all, aligned to the universal health coverage and strive to meet the Sustainable Development Goal 3 for health to curb the access barriers to healthcare. It should be further recommended that there be research on service provision and providers of child healthcare services to get their perspectives on the ubuntu philosophy. As a vehicle for improving child healthcare services access, there is need for orientation of healthcare providers in health institutions to sensitise all on ubuntu values.

## Conclusion

Even though Batho Pele is taught in the South African healthcare training and addresses ‘people first’ values, ubuntu philosophy should be part of the curriculum as it is comprehensive and encompasses Batho Pele in its application. All members of the communities need to be aware that going back to the basics of respect and caring can improve child health and access to childcare services. Insights from this study can be used in crafting child healthcare policies. Preservice and in-service nursing curricular could benefit from incorporating the principles of ubuntu.
